# P-1466. Effectiveness of Nirsevimab in High-Risk Infant Populations: a retrospective observational study following the first season of Chile’s national immunisation strategy (NIRSE-CL)

**DOI:** 10.1093/ofid/ofaf695.1652

**Published:** 2026-01-11

**Authors:** Juan Pablo Torres, Denis Sauré, Miguel O’Ryan, Marcel Goic, Charles Thraves, Jorge Pacheco, Javiera Burgos, Felipe del Solar, Ignasi Neira, Amal Zgheib, Leonardo J Basso

**Affiliations:** Department of Pediatrics, University of Chile, Santiago, Region Metropolitana, Chile; Universidad de Chile & Instituto Sistemas Complejos de Ingenieia (ISCI), Santiago, Region Metropolitana, Chile; Universidad de Chile & Instituto Sistemas Complejos de Ingenieia (ISCI), Santiago, Region Metropolitana, Chile; Universidad de Chile, Santiago, Region Metropolitana, Chile; Universidad de Chile, Santiago, Region Metropolitana, Chile; Ministerio de Salud de Chile, Santiago, Region Metropolitana, Chile; Ministerio de Salud de Chile, Santiago, Region Metropolitana, Chile; Instituto Sistemas Complejos de Ingenieria (ISCI), Santiago, Region Metropolitana, Chile; Instituto Sistemas Complejos de Ingenieria (ISCI), Santiago, Region Metropolitana, Chile; Instituto Sistemas Complejos de Ingenieria (ISCI), Santiago, Region Metropolitana, Chile; Universidad de Chile & Instituto Sistemas Complejos de Ingeniería (ISCI), Santiago, Region Metropolitana, Chile

## Abstract

**Background:**

Importance and objective: Real-life effectiveness of nirsevimab has been reported recently however, effectiveness data on high-risk infants –preterms or/and infants with congenital heart disease (CHD)– is not available.

**Methods:**

Design: We conducted a population-based matched case-control study, where RSV-related LRTI hospitalization cases among high-risk infants were matched at a 1:7 ratio.

Participants: The study included 145,087 infants immunized between April 1st and September 30th 2024, of whom 72,246 (49·79%) were born between October 1st 2023 and March 31st 2024 (the catch-up group), and 72,841 (50·21%) born between April 1st and September 30th 2024 (the seasonal newborn cohort). Preterm infants were identified using birth data from Chile’s official national database, and infants with CHD were identified via relevant ICD-10 codes, from national hospital discharge records. RSV-related hospitalization cases were matched by age, date of hospital admission and geographic region via mathematical integer programming.

Intervention: Single dose of nirsevimab (Beyfortus, Sanofi, Paris, France) administered free of charge to all infants born up to six months before the beginning of the RSV season (April 1st 2024).

Main outcome and measures: RSV-related LRTI hospital admissions determined by ICD-10 codes at hospital discharge.

**Results:**

Nirsevimab's effectiveness in preventing RSV-LRTI hospitalization in all infants was 87·8% (95% CI 85·3% – 89·9%). 248 out of 1,112 cases of RSV-related LRTI hospitalizations were linked to high-risk infants. Cases among preterm infants (n=218) have 33·38 (3.0) weeks of gestational age (wga) and 2·2 (0.7) kgs. of weight at birth on average and were mostly immunized (85·5%). Cases among infants with CHD (n=41) have 35·37 (4·5) wga and 2·6 (1·0) kgs. of weight at birth on average and were mostly immunized (86·5%). Effectiveness of nirsevimab against RSV-related LRTI hospitalizations was 82·4% (95% CI 70·7% – 89·4%) among preterm infants, and 87·88% (95% CI 56·9% - 96·6%) among infants with CHD.

**Conclusion:**

The nirsevimab-based strategy provided high protection to all infants including preterms and those born with CHD. The study provides the first evidence of real-life protection among high-risk infants.

**Disclosures:**

Juan Pablo Torres, MD, PhD, Barath Biotech: Grant/Research Support|MSD: Honoraria|Pfizer: Honoraria|Sanofi: Honoraria Miguel O'Ryan, MD, Barath Biotech: Grant/Research Support Leonardo J. Basso, PhD, Sanofi: Grant/Research Support

